# A predictive model for lack of partial clinical remission in new-onset pediatric type 1 diabetes

**DOI:** 10.1371/journal.pone.0176860

**Published:** 2017-05-01

**Authors:** Katherine R. Marino, Rachel L. Lundberg, Aastha Jasrotia, Louise S. Maranda, Michael J. Thompson, Bruce A. Barton, Laura C. Alonso, Benjamin Udoka Nwosu

**Affiliations:** 1 Division of Endocrinology, Department of Pediatrics, University of Massachusetts Medical School, Worcester, Massachusetts, United States of America; 2 Department of Quantitative Health Sciences, University of Massachusetts Medical School, Worcester, Massachusetts, United States of America; 3 Diabetes Division, Department of Medicine, University of Massachusetts Medical School, Worcester, Massachusetts, United States of America; Baylor College of Medicine, UNITED STATES

## Abstract

**Importance:**

>50% of patients with new-onset type 1 diabetes (T1D) do not enter partial clinical remission (PCR); early identification of these patients may improve initial glycemic control and reduce long-term complications.

**Aim:**

To determine whether routinely obtainable clinical parameters predict non-remission in children and adolescents with new-onset T1D.

**Subjects and methods:**

Data on remission were collected for the first 36 months of disease in 204 subjects of ages 2–14 years with new-onset type 1 diabetes. There were 86 remitters (age 9.1±3.0y; male 57%), and 118 non-remitters (age 7.0±3.1y; male 40.7%). PCR was defined as insulin-dose adjusted hemoglobin A1c of ≤9.

**Results:**

Non-remission occurred in 57.8% of subjects. Univariable analysis showed that the risk for non-remission was increased 9-fold in patients with 4 diabetes-associated auto-antibodies (OR = 9.90, p = 0.010); 5-fold in patients <5 years old (odds ratio = 5.38, p = 0.032), 3-fold in those with bicarbonate of <15 mg/dL at diagnosis (OR = 3.71, p = 0.008). Combined estimates of risk potential for HC0_3_ and the number of autoantibodies by multivariable analysis, adjusted for BMI standard deviation score, showed HC0_3_ <15 mg/dL with a clinically significant 10-fold risk (OR = 10.1, p = 0.074); and the number of autoantibodies with a 2-fold risk for non-remission (OR = 1.9, p = 0.105). Male sex and older age were associated with decreased risk for non-remission. A receiver-operating characteristic curve model depicting sensitivity by 1-specificity for non-remission as predicted by bicarbonate <15 mg/dL, age <5y, female sex, and >3 diabetes-associated autoantibodies had an area under the curve of 0.73.

**Conclusions:**

More than 50% of children and adolescents with new-onset T1D do not undergo partial clinical remission and are thus at an increased risk for long-term complications of diabetes mellitus. A predictive model comprising of bicarbonate <15 mg/dL, age <5y, female sex, and >3 diabetes-associated autoantibodies has 73% power for correctly predicting non-remission in children and adolescents with new-onset T1D. Early identification of these non-remitters may guide the institution of targeted therapy to limit dysglycemia and reduce the prevalence of long-term deleterious complications.

## Introduction

A significant gap in therapeutic management of patients with new-onset T1D is the lack of a distinctive focus on preventing early dysglycemia in children and adolescents who fail to undergo partial clinical remission (PCR), also called the honeymoon phase[1–4]. Type 1 diabetes is a hyperglycemic syndrome resulting from autoimmune destruction of the pancreatic beta cells[5, 6]. The diagnosis of type 1 diabetes is often followed by a partial clinical remission phase which is marked by the recovery of surviving beta cells and increased endogenous insulin production[4, 7, 8]. Residual endogenous insulin secretion in patients with type 1 diabetes is associated with improved long-term glycemic control, reduced risk of severe hypoglycemia[8, 9], reduced risk for the development of diabetic retinopathy[10] and improved statural growth in prepubertal children[11]. A recent long-term study reported a significantly reduced risk for chronic microvascular complications at 7-year follow up in patients who entered PCR [2]. Thus, patients who undergo PCR, also known as remitters, have an overall prognostic advantage over non-remitters, but this is not usually taken into consideration during the early management of children who fail to undergo PCR.

A significant proportion of children and adolescents diagnosed with type 1 diabetes will not experience PCR[1, 12–14]. These children may be at higher risk for the short- and long-term complications of type 1 diabetes[7–10]. The prevalence of non-remission has not been extensively characterized in pediatric patients with new-onset type 1 diabetes, and the risk profile of factors that predict non-remission has not been adequately analyzed. Earlier studies reported a prevalence of non-remission of 61% in adult patients [12], 57% in a combined cohort of pediatric and adult subjects[13], and 60% to 65% in pediatric studies[1, 14]. In one study in adults, age and BMI did not predict PCR, but female sex and low bicarbonate did[12]. However, in another study from the same group, multiple regression analysis showed that PCR in young adults was predicted only by BMI[15]. Autoantibody titer was reported to predict[4, 12] or not predict[16] PCR. Studies in Finnish [17], German and Austrian[4] children and adolescents reported diminished likelihood of remission in children with DKA at the time of diagnosis of type 1 diabetes.

One unexplored area in this field is the determination of the effect of patients’ vitamin D status on remission at the time of diagnosis of T1D. This is important as randomized control trials that examined the role of vitamin D supplementation on PCR[18, 19] suggest that vitamin D supplementation may slow T1D progression[18, 19]. One of these trials [18] reported a significant difference in residual beta cell function between vitamin D group and placebo, while the other [19] did not. Given the lack of consensus on the effect on vitamin D on the duration of PCR from these trials[18, 19]; and the fact that vitamin D has anti-inflammatory and immunomodulatory functions[20], and that vitamin D supplementation could lead to a resolution of inflammatory states[21], we speculated that subjects with vitamin D deficiency at the time of diagnosis of T1D may have an increased risk for non-remission compared to vitamin D sufficient subjects.

To date, available data on the predictors of PCR have been largely derived from studies in adult patients, or mixed adult and pediatric patients[12–14], and some recent pediatric studies[1, 3, 4]. All these studies have focused on characterizing PCR. Thus, the predictors of non-remission in children with new-onset type 1 diabetes and the risk potential of these predictors have not been adequately described. Such characterization would enable endocrinologists to institute measures to ensure optimal glycemic control very early in the course of the disease in non-remitters[3], in contrast to the current treatment paradigm that incorrectly assumes that most patients with new-onset type 1 diabetes undergo PCR. This new approach is important, since available data report a prevalence of non-remission of 39–61% in a mixed population of pediatric and adult patients [12, 15], or as high as 65% in children and adolescents[1, 14]. The frequent occurrence of non-remission, the resulting inadequate early glycemic control, and the data linking non-remission with risk of complications[2] suggest that a detailed analysis of the factors that predict PCR may enable strategies to improve early glycemic control and reduce complications[2, 3]. In addition, early identification of non-remitters would enhance study subject selection or exclusion in beta-cell preservation trials, and ongoing monitoring data following diagnosis of type 1 diabetes for studies aimed at characterizing the prodromal phase of type 1 diabetes such as the TrialNet Consortium.

The aim of this study, therefore, was to identify clinical predictors of non-remission in children and adolescents with new-onset type 1 diabetes. We hypothesized that surrogate clinical markers of diminished residual β-cell function, such as serum bicarbonate at diagnosis, will predict non-remission in these patients.

## Subjects and methods

### Ethics statement

The Institutional Review Board of the University of Massachusetts approved the study protocol and approved the waiver of authorization for the retrospective review of records from patients’ case records. Subjects’ records and related data were anonymized and de-identified prior to analysis.

### Subjects

This study involved the extraction and review of medical records of pediatric patients of ages 2–14 years with a confirmed diagnosis of type 1 diabetes from January 1, 2006 through September 30, 2015 at the Children’s Medical Center Database of the UMassMemorial Medical Center, Worcester, Massachusetts, USA. As detailed in Nwosu et al[22], the diagnosis of type 1 diabetes was based on any of the following glycemic parameters: a fasting blood glucose of ≥ 7 mmol/L (126 mg/dL), and/or 2-hour postprandial glucose of ≥11.1 mmol/L (200 mg/dL), and/or random blood glucose of ≥11.1 mmol/L (200 mg/dL) with symptoms of polyuria and/or polydipsia. Additionally, subjects were positive for one or more diabetes-associated auto-antibodies, including insulin autoantibodies, islet cell cytoplasmic autoantibodies, glutamic acid decarboxylase antibodies, and/or insulinoma associated-2 (IA-2A) autoantibodies. Subjects with other forms of diabetes mellitus were excluded from the study.

Following an initial, standard diabetes diagnostic laboratory blood draw, patients were begun on a standard basal bolus insulin regimen, consisting of a once-daily long-acting insulin and pre-meal short-acting insulin injections, if they were not in diabetic ketoacidosis (DKA). Patients in DKA were started on an insulin drip at 0.05 units/kg/hour and titrated accordingly to maintain glycemia until the resolution of acidosis. All patients were discharged from the hospital on basal bolus insulin regimen. Each Subject’s insulin requirements were expressed as total daily dose (TDD) which is the combined total doses of long-acting- and short acting insulins administered daily, divided by the subject’s weight in kilograms, and expressed in units/kg/day.

In addition to baseline diagnostic data, further anthropometric, clinical (HbA1c, total daily dose of insulin), and biochemical data were collected at baseline at diagnosis, and then every 3 months for the first year, and every 3 to 6 months until 36 months. Missing data were taken into consideration in the statistical analysis using the linear mixed model. DKA was defined by a pH of <7.35, blood glucose of >200 mg/dL, and serum bicarbonate of <15 ng/mL [5]; while PCR was defined by insulin-dose adjusted hemoglobin A1c (IDAA1C), which is a new two-dimensional definition that correlates insulin dose and measured HbA1c with residual β-cell function[23]. IDAA1C has the best correlation with stimulated C-peptide of >300 pmol/L when compared to previous definitions [24]. The formula for IDAA1C is HbA1c (%) + [4 X total daily dose of insulin (units/kg/24h)]. PCR is defined as IDAA1C of ≤9[23]. Vitamin D deficiency was defined as 25(OH)D of <50 nmol/L[25].

### Anthropometry

Body weight was measured using an upright scale to the nearest 0.1 kg. Height was measured to the nearest 0.1 cm using a wall-mounted stadiometer that was calibrated daily. BMI was derived from the formula: weight/height^2^ (kg/m^2^). Height, weight, and BMI data were expressed as standard deviation score (SDS) for age and sex, based on National Center for Health Statistics (NCHS) data.[26] Underweight was defined as BMI of <5^th^ percentile, overweight was defined as BMI of ≥85^th^ but <95^th^ percentile, and obesity was defined as BMI of ≥95^th^ percentile for age and gender.

### Assays

Assay protocol has been previously described[27, 28]. Briefly, serum 25(OH)D concentration was analyzed using 25-hydroxy chemiluminescent immunoassay (DiaSorin Liaison; Stillwater, Minnesota), which measures total serum 25(OH)D content as it detects both metabolites of 25(OH)D: 25(OH)D_2_ and 25(OH)D_3_. It has an intra- and inter-assay coefficients of variation of 5% and 8.2% respectively, and a functional sensitivity of 10 nmol/L. The characterization of vitamin D status for this study was based on The Endocrine Society Clinical Practice Guideline which defined vitamin D status using serum 25(OH)D values as follows: vitamin D deficiency < 20 ng/mL (50 nmol/L), insufficiency 20–29.9 ng/mL (50–74.5 nmol/L), and sufficiency ≥ 30 ng/mL (75 nmol/L)[25]. This characterization is similar to the classification of vitamin D status by the Institutes of Medicine and the American Academy of Pediatrics which denote vitamin D deficiency as 25(OH)D <50 nmol/L; or sufficiency, 25(OH)D >50 nmol/L[29, 30].

Hemoglobin A1c was measured by DCA 2000+ Analyzer (Bayer, Inc., Tarrytown, NY, USA) based on Diabetes Control and Complications Trial standards [31].

Serum bicarbonate was measured at the University of Massachusetts Medical School Clinical Laboratory by total carbon dioxide (C0_2_) estimation using Beckman Coulter AU System C0_2_ Reagent according to the method of Forrester et al[32]. The assays for diabetes-associated autoantibodies were performed by Quest Diagnostics, Chantilly, VA, USA. GAD-65 assay was performed using enzyme linked immunosorbent assay, IA-2A and IAA assays were performed using radio-binding assay, and ICA assay was performed using immunofluorescence technique.

### Statistical analyses

Means and standard deviations (SD) were calculated for descriptive summary statistics and biochemical parameters (Table 1). Linear mixed model was used to compare the means of anthropometric parameters between the remitters and non-remitters. Student’s t test was used to make the comparison between the groups for non-anthropometric continuous variables viz., total daily dose of insulin, and HbA1c[3]; and Fisher exact test was used for categorical variables. Kaplan-Meier estimates were used to investigate the associations between the duration of remission and categorical predictor variables. Combined estimates for risk potential for non-remission were determined by multivariable analysis and adjusted for BMI SDS to account for differences in age and sex (Table 2). The duration of PCR was calculated as the interval between the first and last documented time points with IDAA1C value of ≤9. Logistic regression was used to produce the adjusted receiver-operating characteristic (ROC) curve for remitters. SPSS Predictive Analytics SoftWare v.23 (IBM Corporation, Armonk, NY) and SAS V. 9.4 (SAS Institute, Cary, NC) were used to perform all statistical analyses.

**Table 1:** Comparison of the anthropometric and biochemical characteristics of remitters and non-remitters.

Parameter	Remitters (n = 86)	Non-Remitters (n = 118)	*p*
Age (yr)	9.1 ± 3.0	7 ± 3.1	**<0.001**
Sex (male/female)	49 (57%)/ 37(43%)	48 (41%)/ 70 (59%)	**0.021**
*Height SDS	0.2 ± 1.0	0.3 ± 1.0	0.348
*Weight SDS	0.5 ± 1.0	0.4 ± 0.9	0.587
*BMI SDS	0.6 ± 1.0	0.5 ± 1.0	0.140
Proportion with BMI >85^th^ percentile	25/84 (29.8%)	29/112 (25.9%)	0.549
Proportion with >2 antibodies	25/69 (36.2%)	46/108 (42.6%)	0.400
Duration of PCR (months)	8.8 ± 7.3	0.1 ± 1.1	**<0.001**
pH at diagnosis	7.3 ± 0.1	7.3 ± 0.1	0.116
25-hydroxyvitamin D (nmol/L)	67.5 ± 28.4	66.1 ± 19.4	0.717
HC0_3_ (mmol/L) at diagnosis	21.4 ± 5.9	18.3 ± 8.3	**0.006**
TDD (Units/kg/day) at diagnosis	0.5 ± 0.3	0.5 ± 0.2	0.334
TDD at 6 mo	0.3 ± 0.2	0.5 ± 0.2	**<0.001**
TDD at 18 mo	0.5 ± 0.2	0.7 ± 0.2	**<0.001**
TDD at 24 mo	0.5 ± 0.3	0.7 ± 0.3	**<0.001**
TDD at 36 mo	0.7 ± 0.3	0.8 ± 0.3	0.169
HbA1c (%) at diagnosis	11.4 ± 2.4	11.5 ± 2.1	0.584
HbA1c at 3 mo	7.5 ± 1.0	8.6 ± 1.3	**<0.001**
HbA1c at 6mo	7.3 ± 1.3	8.8 ± 1.3	**<0.001**
HbA1c at 9 mo	7.8 ± 1.0	8.7 ± 1.0	**<0.001**
HbA1c at 12 mo	7.9 ± 1.1	8.7 ± 1.0	**<0.001**
HbA1c at 15 mo	8.1 ± 1.0	8.7 ± 0.9	**<0.001**
HbA1c at 18 mo	8.2 ± 1.1	8.7 ± 1.2	**0.008**
HbA1c at 21 mo	8.4 ± 1.0	8.8 ± 1.1	0.088
HbA1c at 24 mo	8.5 ± 1.1	8.8 ± 0.9	0.068
HbA1c at 27 mo	8.5 ± 1.2	8.5 ± 1.4	0.816
HbA1c at 30 mo	8.5 ± 1.1	8.8 ± 1.0	0.134
HbA1c at 33 mo	8.7 ± 1.4	8.6 ± 1.0	0.721
HbA1c at 36 mo	8.4 ± 1.2	8.8 ± 1.0	0.087

SDS = standard deviation score; BMI = body mass index; TDD = total daily dose of insulin in units/kg/day; 25(OH)D = 25 hydroxyvitamin D; HC0_3_ = bicarbonate; mo = month. HbA1c = hemoglobin A1c

*Comparison made by Linear mixed model. Significant *p* values are bolded.

**Table 2:** Univariable and multivariable logistic model for determinants of non-remission in new-onset pediatric type 1 diabetes.

Variable	Coefficient	SE	Wald	Odds Ratio	95% CI	*p* value
**Univariable Analysis**						
Age^a^ < 3yr	1.68	0.79	4.59	5.38	[1.15–25.10]	**0.032**
Sex (Male)	-0.68	0.29	5.60	0.51	[0.29–0.89]	**0.018**
BMI (kg/m^2^)						
Underweight	0.38	0.69	0.31	1.46	[0.38–5.61]	0.580
Overweight	0.28	0.43	0.41	1.32	[0.57–3.07]	0.522
Obese	-0.58	0.45	1.67	0.56	[0.23–1.35]	0.561
DKA^b^	0.24	0.33	0.54	1.27	[0.67–2.41]	0.461
Number of Diabetes Antibodies^c^						
One autoantibody	1.22	0.78	2.42	3.37	[0.73–15.60]	0.120
Two autoantibodies	1.66	0.78	4.60	5.26	[1.15–24.0]	**0.032**
Three autoantibodies	1.55	0.78	3.91	4.70	[1.01–21.77]	**0.048**
Four autoantibodies	2.29	0.89	6.64	9.90	[1.73–56.56]	**0.010**
Bicarbonate of <15 mg/dL^d^	1.31	0.50	6.95	3.71	[1.40–9.81]	**0.008**
25(OH)D (< 20 nmol/L)	0.12	0.24	0.25	1.27	[0.50–3.28]	0.620
**Multivariable Analysis**						
**Combination** of HC0_3_^d^ of <15 mg/dL and 25(OH)D^d^ of <20 ng/mL						
Bicarbonate of <15 mg/dL^d^	1.13	0.56	3.93	3.04	[1.01–9.16]	**0.048**
25(OH)D^d^ of <20 ng/mL	-0.64	0.51	0.016	0.94	[0.35–2.54]	0.900
**Combination** of HC0_3_^d^ of <15 mg/dL and Auto-antibodies^d^						
Bicarbonate of <15 mg/dL^d^	2.31	1.29	3.19	10.10	[0.80–127.47]	0.074
Auto-antibodies ^d^	0.64	0.40	2.64	1.90	[0.88–4.12]	0.105

^a^ = adjusted for sex

^b^ = adjusted for age and sex

^c^ = adjusted for age

^d^ = adjusted for body mass index (BMI) standard deviation score which incorporates age and sex; DKA diabetic ketoacidosis; significant p values are bolded.

## Results

### A. Baseline analysis

#### Anthropometry

**Fig 1:**
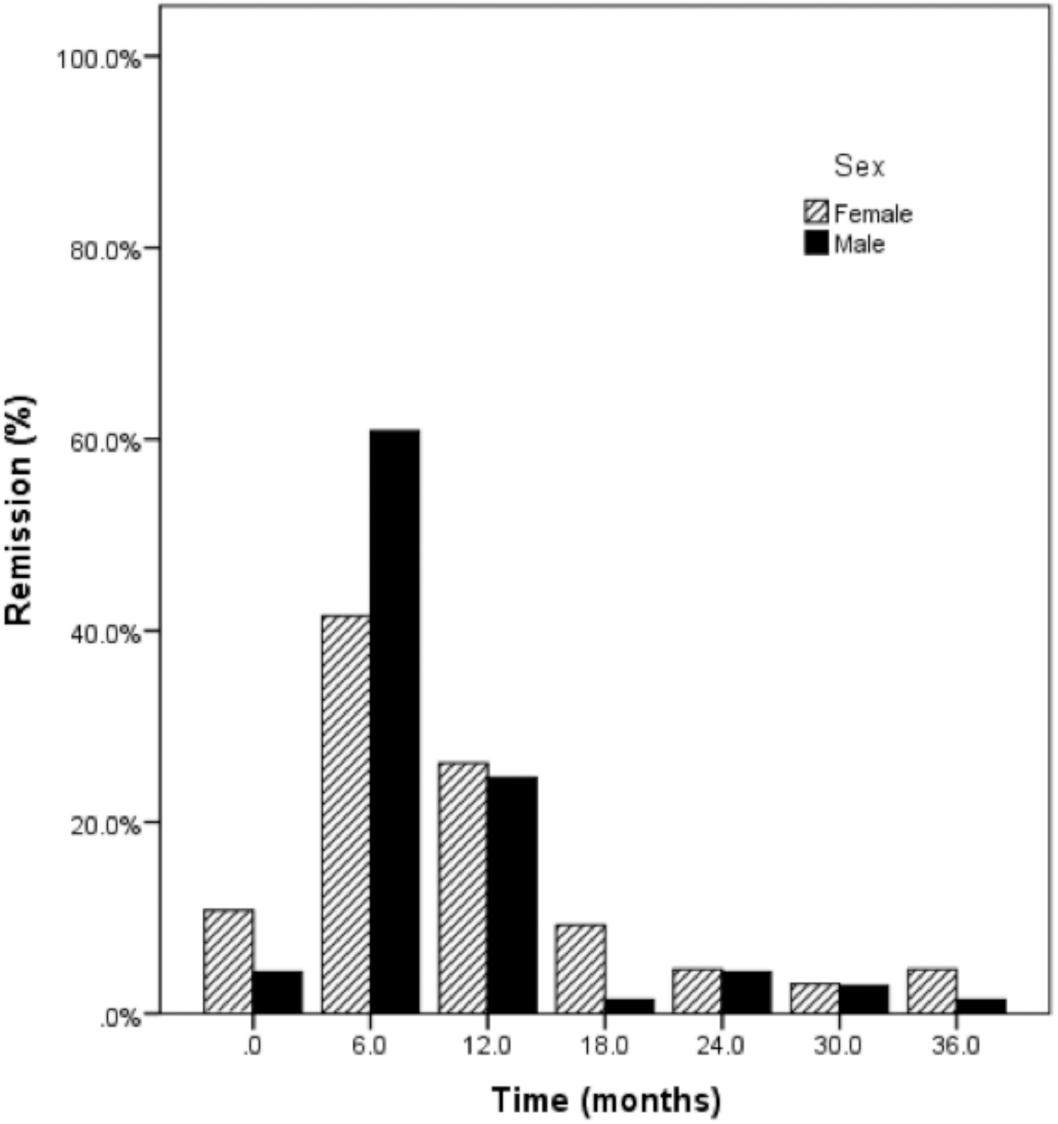
Bar graph of the prevalence of remission at different time points in male and female pediatric subjects with new-onset type 1 diabetes.

Two hundred and four children and adolescents of ages 2–14 years, mean age of 7.9 ± 3.2y, (male 7.8 ± 3.4 yr (n = 98); female 7.9 ± 3.0 (n = 106), p = 0.816) with new-onset type 1 diabetes were analyzed. There were 86 remitters (age 9.1 ± 3.0 y; male 57%), and 118 non-remitters (age 7.0 ± 3.1 y; male 40.7%). (Table 1). The prevalence of non-remission was 57.8%, and the peak period of remission was between 6–12 months (Fig 1).

Non-remitters were younger than remitters (p <0.001), and consisted of more female patients than males. At diagnosis, BMI SDS, which is corrected for age and sex, was similar between the groups (p = 0.414). There was no difference in the proportion of patients with BMI ≥85^th^ percentile (p = 0.549).

#### Biochemical parameters

Of the biochemical parameters evaluated for this study at baseline, only serum bicarbonate (HC0_3_) was significantly lower in non-remitters (18.3 ± 8.3 vs. 21.4 ± 5.9, p = 0.006). There was no difference between the groups for pH (p = 0.116), 25(OH)D (p = 0.717), and HbA1c (p = 0.584). There were no differences in the total daily dose of insulin at baseline between the groups (p = 0.334).

### B. Post-baseline analysis

#### Anthropometry

There was no difference in mean BMI SDS between the groups over the period of study.

#### TDD of insulin

Mean TDD of insulin was significantly higher in non-remitters from 6 months after diagnosis to 24 months. There was no difference in TDD of insulin between the groups at 36 months.

#### HbA1c

**Fig 2:**
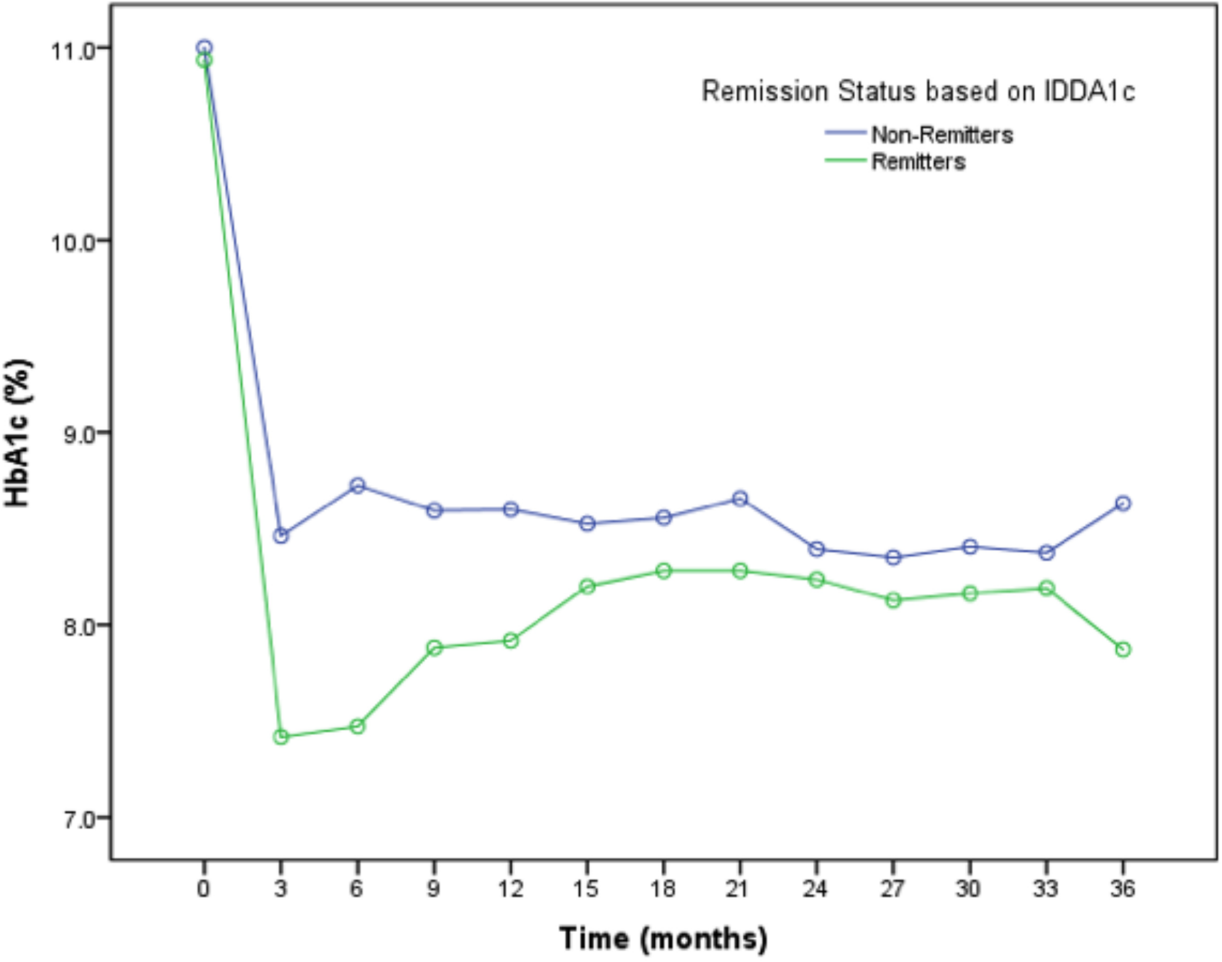
Graphical representation of the pattern of hemoglobin A1c trends in remitters and non-remitters in the first 36 months following the diagnosis of type 1 diabetes. Remission was defined by an insulin-dose adjusted hemoglobin A1c level of ≤9[23]. Mean HbA1c was similar at diagnosis between the remitters and non-remitters 11.4 ± 2.4 vs. 11.5 ± 2.1, p = 0.584, then became significantly lower in the remitters from 3 months, 7.5 ± 1.0 vs. 8.6 ± 1.3, p <0.001, through 18 months, 8.2 ± 1.1 vs 8.7 ± 1.2, p = 0.008, and was non-significantly lower in the remitters thereafter.

Mean HbA1c values were significantly higher in non-remitters from 3–18 months; and then became similar in the two groups from 21 months through 36 months (Fig 2). Specifically, HbA1c was similar at diagnosis between the remitters and non-remitters, 11.4 ± 2.4% vs. 11.5 ± 2.1%, p = 0.584, then became significantly lower in the remitters from 3 months, 7.5 ± 1.0 vs. 8.6 ± 1.3, p <0.001, through 18 months 8.2 ± 1.1 vs 8.7 ± 1.2, p = 0.008, and was non-significantly lower in the remitters thereafter.

### Logistic regression analysis

Logistic regression analysis was used to determine the risk for non-remission associated with each parameter following adjustment for confounders (Table 2). While no specific autoantibody independently predicted the risk for non-remission, there was a 9-fold increased risk for non-remission in patients with 4 diabetes-associated auto-antibodies (OR = 9.90, p = 0.010); 5-fold increased risk for non-remission in patients of <5y (odds ratio = 5.38, p = 0.032), and a 3-fold increased risk for non-remission in subjects with bicarbonate of <15 mg/dL (OR = 3.71, p = 0.008).

As an indicator for potential research, though not statistically significant, the BMI SDS-adjusted combined estimates of risk potential for HC0_3_ and the number of autoantibodies by multivariable analysis showed HC0_3_ <15 mg/dL with a clinically significant 10-fold risk (OR = 10.1, p = 0.074), and the number of autoantibodies with a clinically significant 2-fold risk for non-remission (OR = 1.9, p = 0.105). However, when serum HC0_3_ of <15 mg/dL and serum 25(OH)D of <20 ng/mL were fitted to the model and adjusted for BMI SDS, HC0_3_ of <15 mg/dL conferred a statistically significant 3-fold increase in risk for non-remission, (OR = 3.04, p = 0.048) whereas serum 25(OH)D of <20 ng/mL predicted no increased risk (OR = 0.94, p = 0.900). The reason for adding serum 25(OH)D to the multivariable model for HC0_3_ was to determine whether the anti-inflammatory actions of 25(OH)D had a synergistic or attenuating effect on the risk of HC0_3_ of <15 mg/dL for non-remission following adjustment for BMI SDS as several trials have examined the role of vitamin D supplementation on PCR[18, 19].

In the univariable analysis, male sex, and older age at diagnosis were also associated with decreased risk for non-remission (Table 2). Kaplan-Meier survival curves showed no significant associations between the duration of clinical remission and the predictors of non-remission.

**Fig 3:**
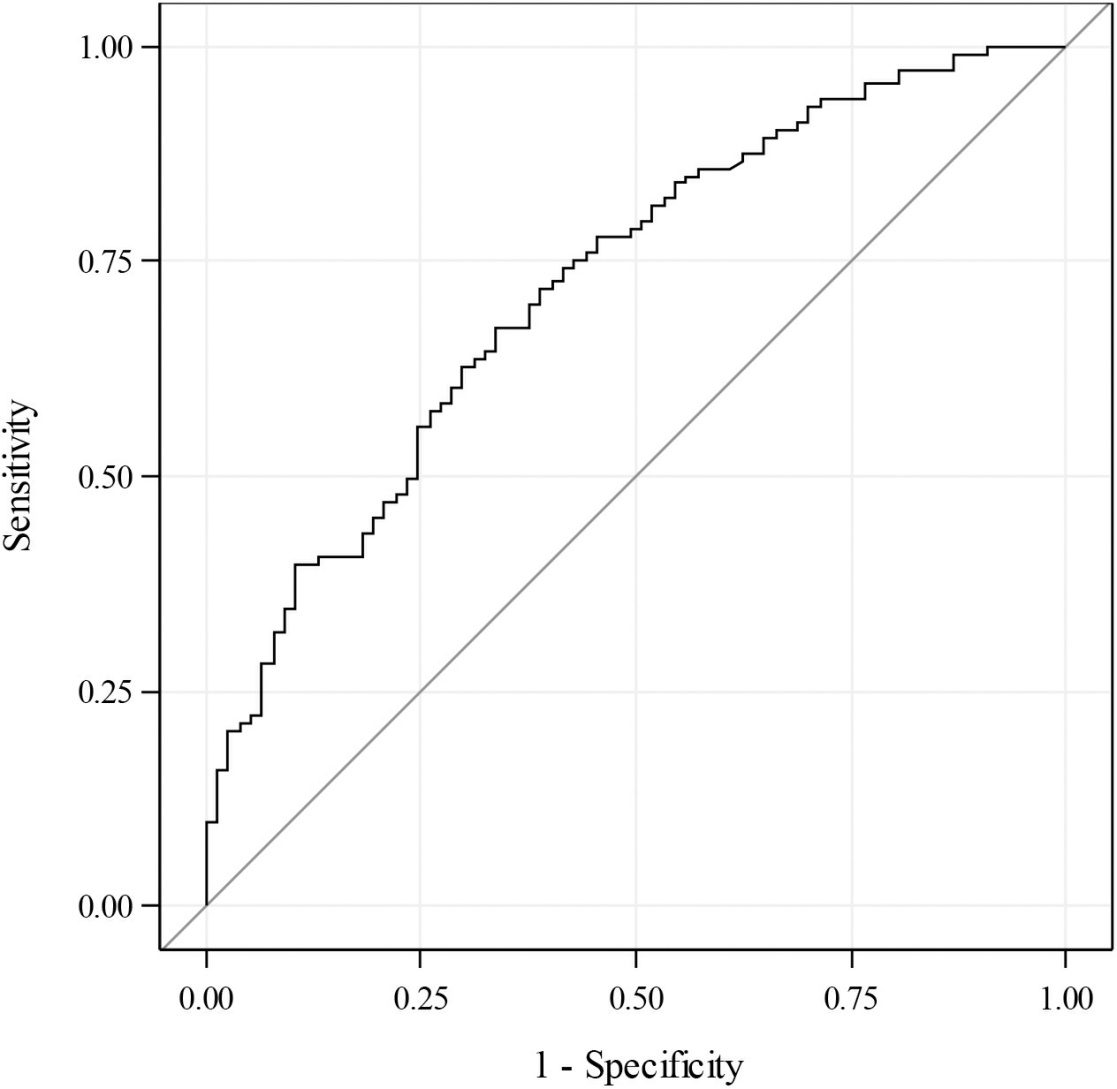
A receiver-operating characteristic curve model depicting sensitivity by 1-specificity for non-remission as predicted by bicarbonate <15 mg/dL, age <5y, female sex, and >3 diabetes-associated autoantibodies. The area under the curve is 0.73.

Fig 3 shows the receiver-operating characteristic (ROC) curve depicting the sensitivity by 1-specificity for the final model for non-remitters as predicted by serum bicarbonate <15 mg/dL, age <5y, female sex, and >3 diabetes-associated autoantibodies. This ROC curve had an area under the curve of 0.73. This indicates that this model, which combines bicarbonate, age, sex, and number of antibodies correctly predicted the remission status of 73% of the patients with new-onset type 1 diabetes as either non-remitters or remitters.

## Discussion

**Fig 4:**
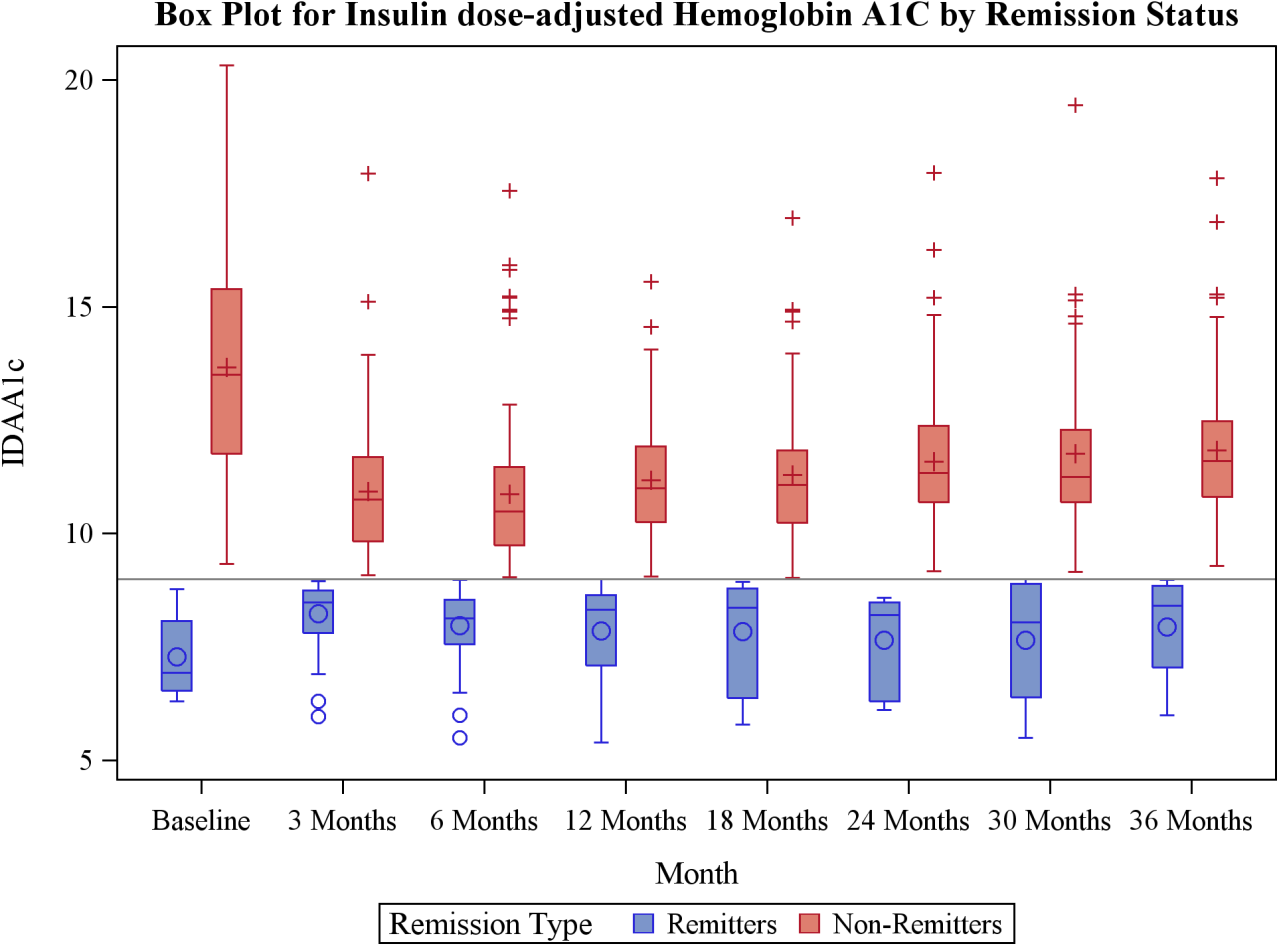
Box plot for the change in insulin dose adjusted hemoglobin A1c level over time by remission status.

This is the first detailed characterization of the predictors of non-remission, and their risk potential in children and adolescents using longitudinal IDAA1C measurements over a 3-year period (Fig 4). Clinical data indicate that patients with newly-diagnosed type 1 diabetes who undergo PCR have persistent C-peptide level, improved glycemic control in the short term (Fig 2), and reduced prevalence of diabetes complications in the long-term[2, 33]. However, up to 60% of patients with new-onset type 1 diabetes are non-remitters, and thus are not expected to experience these advantages in the life history of their disease[12, 13, 16]. Our dataset quantifies the glycemic cost of non-remission: in our cohort the HbA1c was significantly higher in non-remitters than remitters for an extended period spanning 3–18 months post diagnosis. Simple clinical parameters that indicate increased risk for non-remission are not fully characterized and there is no uniform strategy to identify these patients and prevent early dysglycemia that may have negative consequences later in life[2].

This study examined the prevalence and key indicators of non-remission in children with new-onset type 1 diabetes. It found that the prevalence of non-remission was 57.8% in children and adolescents with new-onset type 1 diabetes. It also determined that serum bicarbonate of <15 mg/dL, along with increasing number of diabetes-associated autoantibodies, female sex, and young age at diagnosis were the principal predictors of non-remission in this population. In contrast, male sex, and older age, were associated with decreased risk for non-remission. Serum 25(OH)D appeared to have no effect on the risk of non-remission.

The findings from this study are in agreement with data from previous studies in both children and adults. For example, the prevalence of non-remission of 57.8% found in this study is similar to the 61% described in adult patients by Scholin et al[12], 57% described in a combined cohort of pediatric and adult subjects[13], and 60% to 65% in pediatric studies[1, 14]. The anthropometric findings are also in agreement with earlier reports that decreased BMI values were associated with increased risk for non-remission[15], and that female subjects have a higher frequency of absent or shorter duration of remission than male subjects[4, 12]. The higher frequency of non-remission in female patients compared to male patients is believed to be due to several factors including the fact that male subjects have lower islet cell antibody positivity and less aggressive disease course than female subjects[12]. The more aggressive disease phenotype in female subjects is supported by a report of a lower C-peptide concentration in female subjects at the time of diagnosis of type 1 diabetes[34].

Our findings are also in agreement with an earlier report indicating that the presence of increasing number of diabetes-associated autoantibodies is associated with non-remission or shortened PCR[15], but differs from studies reporting specific risk potential with a particular autoantibody, such as the report that higher levels of islet autoantibodies are associated with rapid development of beta-cell failure and low C-peptide concentration[35, 36].

Our finding of significantly increased risk for non-remission at serum HC0_3_ of <15 mg/dL, an index of DKA, is consistent with an adult study showing that low serum bicarbonate of <20 mg/dL at diagnosis is associated with a lower frequency of PCR[12], as well as reports of diminished residual beta cell function in children with DKA at the time of diagnosis[25]. In contrast to the significant difference in serum HC0_3_ between the remitters and non-remitters, there was no significant difference in pH of <7.35 or DKA diagnosis between the remitters and non-remitters. This may relate to the broader definition of DKA; although 98% of subjects with serum bicarbonate of <15 mg/dL had DKA, 9.9% of subjects with serum bicarbonate of >15 mg/dL were still diagnosed with DKA based on pH <7.35, blood glucose of >200 mg/dL, and severity of clinical presentation. Thus, it is possible that serum HC0_3_ of <15 mg/dL is more sensitive than pH of <7.35 or DKA diagnosis for the detection of acidosis and diminished residual β-cell function.

The mechanism of induction of non-remission is not clearly understood but is believed to be associated with increased beta-cell strain. Published studies suggest that PCR is associated with improved processing of proinsulin[16], a finding that has been described in male subjects and those with higher BMI. It has also been proposed that patients who undergo PCR may have a cytokine profile with less damaging effect on the β-cells[37]. Finally, PCR has been associated with lower glucagon concentration, which is consistent with the finding that glucagon production is suppressed by intra-islet insulin release[38].

This study has several limitations that should be taken into consideration in the interpretation of the results. This was a cross-sectional study, and thus, no causality should be inferred regarding the parameters studied and their outcomes. This sample size was relatively small and could have limited the detection of subtle differences between the groups. The study was conducted in one tertiary institution in a particular geographic location, latitude 42°N, and thus the results may not be generalizable to other locations. The strengths of the study include the use of IDAA1c of ≤9 to define PCR, as this new definition has been validated to correlate insulin dose and measured HbA1c with residual β-cell function[23]. Additional strengths include the use of a representative sample of both remitters and non-remitters that allowed meaningful comparisons of the differences between the groups. Thirdly, data collection extended up to 36 months, a period long enough to encompass the usual duration of PCR.

## Conclusions

More than 50% of children and adolescents with new-onset T1D do not undergo partial clinical remission and are thus at an increased risk for long-term complications of diabetes mellitus. A predictive model comprising of bicarbonate <15 mg/dL, age <5y, female sex, and >3 diabetes-associated autoantibodies has 73% power for correctly predicting non-remission in children and adolescents with new-onset T1D. In contrast, male sex, and older age were associated with decreased risk for non-remission, while serum 25(OH)D had no effect on the likelihood of non-remission. Early identification of these non-remitters may guide the institution of targeted therapy to limit dysglycemia and reduce the prevalence of long-term debilitating complications of type 1 diabetes.
